# Building Community Partnership to Sustain the Minnesota Student Parent Support Initiative

**DOI:** 10.1007/s10995-020-02958-8

**Published:** 2020-06-04

**Authors:** Sheila E. Amenumey, Elizabeth A. Gardner, Kathryn M. Linde, Amy L. Margolis

**Affiliations:** 1 Minnesota Department of Health, Child and Family Health Division, Maternal and Child Health Section, P.O. Box 64882, St. Paul, MN 55164-0882 USA; 2 Department of Health and Human Services, Office of the Assistant Secretary for Health Office of Population Affairs, 1101 Wootton Parkway, Suite 700, Rockville, MD 20852 USA

**Keywords:** Sustainability, Community Partnership, Office of Population Affairs, Expectant and Parenting Students, Postsecondary Student Parents

## Abstract

**Purpose:**

Considerable attention has been given to the sustainability of adolescent health programs as federal funds have become limited. This article describes important steps and lessons learned in seeking buy-in from stakeholders to promote sustainability and secure non-federal funds to maintain the Minnesota Student Parent Support Initiative (MSPSI) after federal funding ended.

**Description:**

MSPSI was established in 2010 to address the academic and health needs of expectant and parenting postsecondary students. MSPSI provided coordinated case management and referrals to health, education, and social services for expectant and parenting adolescents, as well as for their children, through Student Parent Centers (SPCs). Six important actions sustained the SPCs after the Office of Population Affairs (OPA) grant funds ended in November 2017: (1) preparing and planning for sustainability, (2) creating and engaging a sustainability committee, (3) assessing sustainability needs and creating a sustainability plan, (4) creating a data system to collect relevant data, (5) building capacity to support communication with decision makers, and (6) sharing data and success stories.

**Assessment:**

The implementation of the sustainability plan resulted in ongoing communications and data sharing with key partners that helped secure additional funds for continuing the program after OPA funding ended.

**Conclusion:**

Implementing the MSPSI sustainability plan developed from OPA’s sustainability framework was effective in sustaining the SPCs after federal funding ended. The sustainability planning, the ability to secure funds, the attempt at passing legislation, and the lessons shared in this article provide valuable guidance to organizations seeking strategies to sustain adolescent health programs.

## Significance

*What is already known on the subject*: Much emphasis has been placed on assessing the efficacy of adolescent health programs, but little attention has been paid to sustaining the programs after federal funding ends. To our knowledge, there has been no comprehensive discussion in literature on how building community partnerships and early planning can sustain adolescent health programs, particularly student parent programs.

*What this study adds*: This paper illustrates how selecting the right stakeholders, early sustainability planning, and development of strategic steps could be useful for sustaining adolescent health programs.

## Purpose

Expectant and parenting college and university students are part of a growing population of nontraditional students, and they are often referred to as postsecondary student parents (PSSP). According to a National Postsecondary Student Aid Study, 22% of all college students are parents, and the majority (51%) of PSSP are students of color, compared with students without children (Reichlin et al. 2019). Research shows that parenting and financial demands often prevent PSSP from attending school or completing their academic and self-sufficiency goals (Schatzel et al. 2011; Miller-Brown 2002; Musu-Gillette et al. 2016; Jensen 2011; Noll et al. 2017; Gault et al. 2014; Goldrick-Rab and Sorensen 2010). Given that a greater proportion of PSSP are financially independent, this student population faces considerable challenges balancing schoolwork, employment, financial pressures, child care arrangements, and family life (Smith 2010; Miller 2010; Carter et al. 1994; Demby et al. 2014).

Although student parents face numerous challenges, successfully completing postsecondary education has a lasting effect on both the student and children (Hartmann and Hayes 2013; Jensen 2011; Noll et al. 2017; Goldrick-Rab and Sorensen 2010). Higher academic achievement yields positive outcomes such as higher wages, better living conditions, greater access to resources, and increased chances of their children pursuing higher education (Hartmann and Hayes 2013; Noll et al. 2017). Given the benefits associated with the educational achievement of PSSP, it is critical that funds are made available to support services that target expectant and parenting college students.

The Minnesota Student Parent Support Initiative (MSPSI) was funded through the Pregnancy Assistance Fund (PAF) authorized through the 2010 Patient Protection and Affordable Care Act (2010). In 2010, the Minnesota Department of Health (MDH) received PAF funds through the Office of Population Affairs (OPA), formerly the Office of Adolescent Health, to improve the health, education, social, and economic outcomes of PSSP and their families (Office of Adolescent Health 2010). Two MSPSI grant goals were (1) expectant and parenting teens, women, and men accomplish their postsecondary education goals at an institute of higher education (IHE) and (2) expectant and parenting teens, women, and men maintain positive health and well-being for themselves and their children.

The Minnesota Office of Higher Education (MOHE) reported that, during the 2014–2015 academic year, 31,392 undergraduate students across Minnesota were parents (Djurovich et al. 2016). To support student parents, MDH provided grants to nine IHEs to establish or expand Student Parent Centers (SPCs). The SPCs were strategically located on IHE campuses to help address the essential needs (e.g., health, parenting, child development, vocational training, and social service application assistance) of PSSP either through direct assistance or by referring PSSP to campus or community supports and resources. SPCs’ practices for interventions with student parents were theory based and evidence informed. Based on gaps in student supports and knowledge of best practices and promising strategies in maternal and child health, MSPSI developed services for PSSP in postsecondary educational settings (Smith 2010). In addition to other grant obligations, the OPA required all the PAF grantees to participate in program sustainability planning (Warner et al. 2020).

Increasingly, funding agencies and organizations are confronted with the challenge of distributing scarce resources. More funding agencies are requiring grantees to plan early for sustainability and to identify strategies for sustaining programs after grant funding ends (Sandy and Holland 2006; Israel et al. 2006; Seifer 2006; Warner et al. 2020). Several articles have explored the importance of engaging community partners to develop and sustain public health programs (Cowan et al. 2004; Austin et al. 1999; Peters et al. 2005; Buys and Burnsnall 2007; Sandy and Holland 2006; Vernon and Foster 2002; Seifer 2006). To our knowledge, there is no comprehensive discussion on how state agencies can collaborate with universities and colleges to build, operate, and sustain partnerships that are valuable in maintaining expectant and parenting adolescent health programs.

At the early stages of MSPSI program implementation, MDH sought to develop a comprehensive and robust implementation process for the future sustainability of the program. MDH invited community partners to collaborate with SPC staff to prepare and implement a sustainability plan for MSPSI. This paper discusses how MDH engaged multiagency partners, including colleges, universities, and community organizations, to sustain the SPC. The purpose of this article is to share lessons learned during the development and implementation of a plan to sustain MSPSI and to describe how the process was beneficial in maintaining SPCs after OPA’s funding ended in 2017.

## Description

The focus of MSPSI was to improve health outcomes and educational attainment for PSSP enrolled at the nine colleges and universities with SPCs. Through the SPCs, MSPSI staff provided coordinated case management and referrals to health, education, and social services for PSSP as well as for their children.

SPC staff offered free and voluntary educational assessments (academic level assessment); academic needs assessment (tutoring options, resource needs, laptops, financial aid); and health screenings (depression, intimate partner violence, tobacco and alcohol). Upon completing health screenings and educational needs assessments, the SPC staff referred PSSP to campus or community-based health (mental health, reproductive health, vision/hearing screening); education; and social (child care subsidies, Medicaid) services. Furthermore, the SPC provided direct or referral services to material supports (food, clothing) and child health supports (lactation programs, well child visits, child care) to PSSP, their children, and immediate family members. The SPC staff also hosted parent education classes, workshops, and other activities designed to meet participants’ health and education needs.

Maintaining the operations of the SPC required staff with expertise from various professional backgrounds. Social workers, counselors, psychologists, and program coordinators with knowledge of social services or higher education served as the SPC coordinators or managers. MDH planned and hosted more than 15 professional development training courses (e.g., in-person workshops, webinars) based on biannual surveys that assessed the SPCs’ staffs’ training needs. MDH offered a variety of training courses on health, education, and social service topics to help staff develop all the necessary skills required to serve PSSP and promote the services offered at the SPC. All SPCs were required to send at least one staff member to each professional development training. If an SPC’s staff missed a training, MDH distributed the accompanying handout materials.

**Fig. 1:**
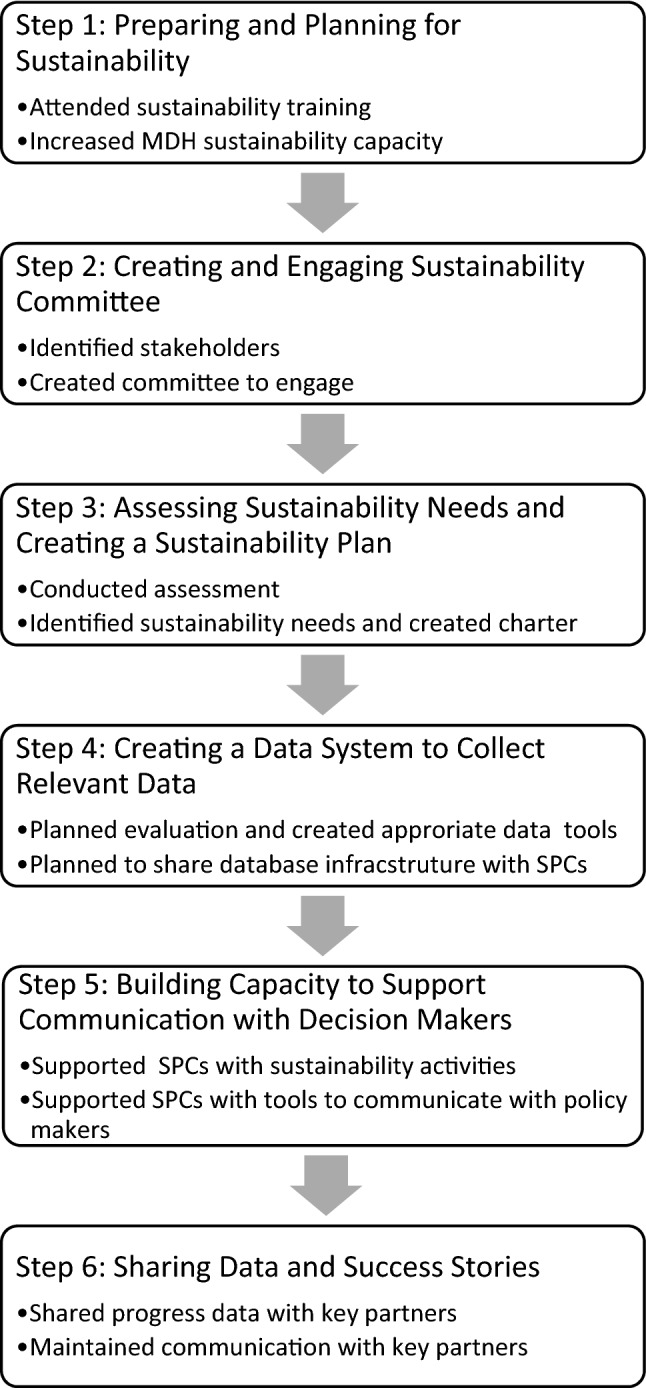
MSPSI sustainability process

At the end of the 2016–2017 academic year, the majority of participants had plans to reenroll for classes and to continue receiving services through the SPC. However, OPA’s grant funding was to end in November 2017. The strategic activities to support SPC sustainability we discuss here were very meaningful in maintaining the SPC through funds secured primarily from MOHE after OPA’s grant funding ended. We identify six critical steps (Fig. 1) that helped in securing funds for sustaining the SPC. Implementing a statewide public health program while pursuing its sustainability was a dynamic process. Although these six steps are presented as if they occurred in sequential order and isolation, they overlapped and the process was not always linear. We draw several lessons from implementing the six steps that may be helpful to others interested in creating a sustainability process to sustain adolescent health programs.

### Step 1: Preparing and Planning for Sustainability

In November 2012, MDH defined its preliminary role with the IHEs for sustaining the program. For example, MDH encouraged IHEs to have conversations with internal and external decision makers. MDH also offered to write letters of support for IHEs’ grant applications to alternative funders and distributed a list of websites and toolkits dedicated toward sustaining college programs for postsecondary at-risk students.

The sustainability process officially began with MDH staff attending a sustainability workshop held by OPA in March 2014 to support grantees’ development of a program sustainability plan. The following OPA definition guided MSPSI’s sustainability planning:

Sustainability is effectively leveraging partners and resources to continue programs, services and/or strategic activities that result in improvements in the health and well-being of adolescents and young adults. (U. S. Department of Health and Human Services 2013)

After the March 2014 OPA sustainability workshop, MDH became more structured and intentional in its approach to program sustainability. To ensure the success of the MSPSI sustainability plan, MDH started the process of identifying key stakeholders to champion the initiative.

### Step 2: Creating and Engaging Sustainability Committee

Stakeholder engagement plays an essential role in creating and sustaining public health programs (Demby et al. 2014). As potential stakeholders were considered, MDH consulted and invited decision makers from IHEs, local public health agencies, and state agencies to join the Sustainability Committee. MDH identified and categorized stakeholders according to their interest levels (Bryson 2004). Additionally, stakeholders were engaged in the sustainability process according to their knowledge of, expertise in, and political power related to the health and educational needs of PSSP. For instance, MDH invited MOHE to join the Sustainability Committee because of its primary responsibility for developing and advocating for a state legislative agenda that financially supports the needs of college-age students, including PSSP. These committee members also shared expertise in financial aid and child care grant programs. The Minnesota Department of Human Services was invited to join the committee; its buy-in was important because the department manages the Minnesota Family Investment Program (i.e., Minnesota’s Temporary Assistance for Needy Families program) and has deep knowledge about PSSP eligibility and employment and training requirements. The Department of Employment and Economic Development was invited to engage with the committee because of its expertise in youth training and vocational development programs. Additional information about the organizations that sent representatives to the Sustainability Committee and their roles is listed in Table 1. Sustainability Committee members were recruited from May through July 2014, and MDH hired a facilitator to organize and facilitate all of the committee’s meetings. Recruiting committee members and hiring a facilitator required three to four months for planning and implementing the necessary activities. The committee met five times between August 2014 and December 2014. The relationships that resulted between these multiple agencies and organizations serving expectant and parenting youth and the SPCs were invaluable in the sustainability process.

**Table 1:** Stakeholder types and expertise

Organizations representing the sustainability committee	Division/program(s)	Role of representatives^a^
Minnesota management and budget	Management analysis and development division	Co-designed planning process with MDH Co-planned meetings’ activities with MDH Typed meeting minutes Facilitated meetings and summarized key findings
Minnesota department of human services	Economic assistance and employment support^b^	Shared details about employment and training requirements for the Minnesota family investment program (minnesota’s temporary assistance for needy families program) Shared eligibility information on supplemental nutrition assistance program and child care subsidies
Minnesota office of higher education	Commissioner’s office, research and policy division and the financial aid division	Shared expertise about financial aid and child care grant programs Facilitated process to secure state funds for MSPSI Provided technical assistance about legislative proposal
Department of employment and economic development	Youth training and employment programs	Facilitated communication between SPCs and local workforce centers Shared information on youth employment programs
Minnesota department of health	Child and family health division, maternal and child health section	Selected committee members Convened committee and drafted program charter Selected a neutral committee facilitator Served as fiscal agent and technical assistance advisor to IHEs Led the evaluation design and implementation and outcome reporting activities Wrote program sustainability report for OPA
Institutes of higher education Century college Fond du Lac Tribal and Community college Leech Lake Tribal College Metropolitan State University Pine technical and community college Riverland community college St. Catherine University St. Cloud State University Winona State University	Varied; some student Parent centers housed in student affairs departments, some in student activities	Advised sustainability committee about the significance of student retention rates as a seminal indicator for decision makers Articulated the social, academic, financial, and health needs of PSSP Described the unique and pressing needs of students of color and American Indian students Explained how student parent centers’ services benefitted the children of PSSP Spoke to the significance of having a student parent program participant as a program sustainability committee member
Postsecondary student parents	Student parent representatives from the University of St. Catherine	Explained the needs of student parents

^a^All committee members collectively defined the vision of the sustainability committee and identified community partners to expand the student parent program. The committee was also involved in identifying additional indicators beyond those required by the office of population affairs

^b^Represented various programs that served student parents (e.g., temporary assistance to needy families, supplemental nutrition assistance program, medicaid, child care subsidies, and other social services programs)

### Step 3: Assessing Sustainability Needs and Creating a Sustainability Plan

MDH assessed the nine IHEs about community needs and their readiness to embark on sustainability activities. A sustainability survey also assessed IHEs’ sustainability planning activities to identify areas for improvement. The sustainability tool was adapted from OPA’s Sustainability Framework Guide (U.S. Department of Health and Human Services 2013). The results of the sustainability assessment identified the strengths of each IHE to engage in sustainability activities. The committee’s project charter was drafted using information from the survey and OPA’s framework. By developing a project charter, shared with committee members with the invitation for the first meeting, MDH defined the scope, purpose, and deliverables of the work. The committee used the survey results to make recommendations, identify sustainability strategies, and plan activities that aligned with the purpose described in the project charter.

The Sustainability Committee prepared a plan describing the committee’s sustainability activities, goals, and strategies, which were embedded in the Sustainability Plan OPA required the grantees to submit. The Sustainability Plan provided a framework for implementing program sustainability activities: (1) secure alternative funding sources and continue the PSSP program on the nine IHE campuses after federal funding ended; (2) open SPCs at additional community colleges, technical colleges, and private and public universities; (3) connect SPCs statewide to advocate for PSSP; (4) increase public, employer, and legislature awareness of the barriers PSSP face; and (5) create infrastructure to conduct research studies on the needs of PSSP. The strategic activities of the committee informed other agency staff of the essential needs of PSSP, which led to opportunities to identify continuation funding.

### Step 4: Creating a Data System to Collect Relevant Data

Data collection was an integral part of the sustainability process because it ensured relevant information was gathered to evaluate activities and advocate for funding to sustain the SPCs. The sustainability activities discussed in this paper were not based upon clinical study or patient data.

MDH ensured all data privacy protocols were met before proceeding with data collection. The MSPSI team obtained approval from MDH’s Institutional Review Board (IRB) to proceed with MSPSI as an evaluation of a public health program. Participating IHEs submitted letters of agreement to MDH indicating they were working with their respective IRBs.

MDH created a data system to support individual SPC evaluation and program sustainability strategies. All SPCs used the same data system to collect OPA required performance measures and other information suggested by the Sustainability Committee. The data systems included four data tools (Table 2) designed to meet OPA and the Sustainability Committee data collection expectations.

**Table 2:** MSPSI data tools and methodology

Data tools	Inclusion criteria	Measures	Data collection method	Frequency of data collection	Staff responsible
Intake form	New PSSP	Baseline demographics	Administered at intake	Collected data at intake, updated as needed	Administered by SPCs Analyses by MDH staff
MSPSI database	All enrolled PSSP at IHE sites	Demographics pregnancy/parenting status, family, academic indicators, health indicators, program activities and referrals	Program records extracted from official academic records included in MSPSI database	Data collected periodically as each participant’s activities and services recorded throughout the semester	Data reporting by staff Data analyses by MDH staff
Student Parent Experience Questionnaire	All enrolled PSSP at IHE sites	Attitudes, knowledge, and behavior of current MSPSI participants	Administered in person or online	Administered at end of fall and spring semesters	Administered by SPCs Data analyses by MDH staff
Annual capacity assessment	All SPCs	Current status of program services and skills related to program sustainability	Administered online	Administered during the fall and spring semester	Administered by MDH Data analyses by MDH staff

#### Intake Form

New participants completed the intake form during enrollment. The form allowed the SPCs to collect baseline data such as demographics, family structure, living situation, and academic information. The information collected on the intake form was incorporated into the MSPSI database. This baseline information provided a foundation for monitoring student progress and the effectiveness of MSPSI that supported the efforts to sustain the program.

#### MSPSI Database

MDH designed the MSPSI database to track program participants longitudinally, to enhance its ability to evaluate relationships between program measures and outcomes. The database automatically generated identification numbers for all participants across the nine IHEs. The unique identification numbers assigned to participants helped track their progress and departures from the program, their employment status, high school or General Education Diploma status, screenings and referrals, support services, and reenrollment during the grant period without personally identifying the student. More and more, decision makers are demanding that programs demonstrate how they are improving the lives of people they serve and how these changes have a real impact on their communities. The ability to track program participants helped MDH and SPCs use data to inform stakeholders and future funders about the impact of the program.

MDH staff analyzed the data from the MSPSI database using SAS software (SAS Institute Inc. 2011) to create aggregate reports for each IHE and across sites to share with their partners and potential funders. During the early stages of the database design, MDH strategically decided to share the database infrastructure and application with each SPC after OPA’s funding ended. After OPA funding ceased in November 2017, MDH with Minnesota Information Technology (MNIT) Services transferred IHEs’ data and the software application to the nine SPCs for their use. Consequently, the nine IHEs each received a jump drive with their own data files and the database software application to continue evaluating the program. MDH and MNIT Services staff worked with some of the IHEs’ information technology staff to load and install the database application software on their servers to safeguard the security of the data shared. The transfer of the data system software to the IHEs ensured SPCs’ evaluation activities continued after OPA funding ceased.

#### Student Parent Experience Questionnaire

The Student Parent Experience Questionnaire measured MSPSI participants’ satisfaction, attitudes, and services received. The survey responses assisted in evaluating the effectiveness of the program services in helping participants reach their academic goals and improve their health and well-being. The feedback received from the participants helped the SPCs demonstrate the program’s impact.

#### Capacity Assessment

The capacity assessment required the SPCs to identify current partners, barriers, and needs concerning serving PSSP participants at the beginning (pre) and end (post) of each grant year. Analyzing the pre and post assessments helped identify the SPC’s progress and barriers to sustainability. MDH used the capacity assessment results to organize training about basic program sustainability concepts to increase the SPCs’ staffs’ capacity to engage in sustainability activities.

### Step 5: Building Capacity to Support Communication with Decision Makers

MDH and the SPCs hosted several multipurpose training events to build SPCs’ staff’s capacity to discuss alternative funding solutions with their institutions’ decision makers and other prospective funders, including private organizations. MDH trained SPC staff on OPA’s eight sustainability factors and framework (U.S. Department of Health and Human Services 2013). In addition, MDH hosted two different training courses about communicating organizational success with brief “elevator speeches” and social media techniques. MDH provided various tools, guidelines, and materials to help grantees communicate effectively with decision makers. For example, Minnesota’s U.S. congressional delegation and the Minnesota Legislative Office on the Economic Status of Women inquired about the health and education needs of expectant and parenting students and MSPSI’s outcomes. In response, MDH prepared fact sheets describing the number of PSSP and children served and the supports provided by the grant. MDH also offered a list of the colleges and universities operating SPCs and shared it with the Minnesota Legislative Office on the Economic Status of Women. These capacity building activities were meaningful because the SPC staff increased their sustainability knowledge and communication skills, and they ultimately spearheaded the implementation of their sustainability strategies. Having received the necessary training, tools, and resources from MDH, the SPCs communicated with multiple organizations with interests in promoting adolescent health about the value of MSPSI and the need for alternative funding.

### Step 6: Sharing Data and Success Stories

SPCs used several strategies to tell the story of their program participants’ activities and accomplishments. For example, MDH shared qualitative and quantitative evaluation reports regularly with all nine SPCs’ staffs to help disseminate their success stories. Using the data received from MDH, the SPCs created success stories describing the student parents’ participation in the respective SPCs and their academic accomplishments. One SPC secured ongoing funding directly through the university because it shared data and success stories demonstrating the academic progress of PSSP with the university president and academic board.

SPC staff not only shared data about PSSP participation, but they also shared the program’s impact to decision makers in their communities. For example, during the final calendar year of MSPSI, staff from one urban university communicated frequently with decision makers about its SPC’s positive impact using data. An SPC coordinator, who was skilled at describing statistics and sharing students’ individual success stories, described the positive qualitative and quantitative data results about graduation and reenrollment. Consequently, the university’s decision makers, such as the dean of students and the provost, received frequent updates on the center’s role in student retention and community building. Also, the SPC coordinator actively participated in the university’s budget planning process and advocated for the institutionalization of the SPC into the university’s overall 2017–2018 budget. Eventually, this SPC secured funding from its university because it shared data and success stories and participated in the budget process.

Furthermore, MDH and SPCs intentionally communicated with MOHE about MSPSI outcomes, to inform the office that had primary responsibility for developing and advocating for a state legislative agenda that financially supported the needs of college students. These connections contributed to MOHE’s decision to allocate $687,000 in state funds to maintain operations at eight SPCs from August 1, 2017, through June 30, 2018. The ninth SPC did not apply for these funds because it received funding from its university.

In 2017, the Minnesota Legislative Office on the Economic Status of Women introduced a bill to the Minnesota House of Representatives for the ongoing support of SPCs. MDH collaborated with MOHE to provide technical assistance for this legislation, offering technical support, progress data, and language needed for successful legislative hearings. Minnesota legislators heard the bill, and SPC staff and students were asked to testify during committee hearings. The SPC staff ensured that the PSSP were well prepared to share their own stories at committee hearings when necessary. The Minnesota House of Representatives voted on the bill, but it lacked the majority votes to pass. Although the bill did not pass, the opportunity to share the SPCs’ and PSSP success stories during the legislative hearings raised the awareness of the program. Data and storytelling were integral parts of MSPSI sustainability because they increased the awareness of the program among potential funders of the SPCs.

## Assessment

In this section, we discuss the lessons learned during the implementation of the sustainability plan that helped secure additional funds for continuing the expectant and parenting student programs after OPA funding ended in 2017.

### Lessons Learned

#### Identifying the Right Stakeholders Early

Beginning the sustainability planning and activities at an early phase of program implementation was beneficial because it allowed enough time to leverage and engage partnerships for sustainability. Recruiting and keeping stakeholders engaged from the inception of the MSPSI sustainability process was time consuming but had numerous benefits. Including the right members on the Sustainability Committee was critical in building a community partnership that understood the needs of PSSP. Selecting stakeholders from multiple agencies and organizations based on their interest and political influence, knowledge of, and expertise in the health and educational needs of PSSP proved to be effective. The opportunities the SPCs had to connect with multiple organizations prompted continuing conversations culminating in identifying additional sources of funding.

#### Instituting a Formal Stakeholder Engagement Process

Establishing a formal process for selecting and setting up the Sustainability Committee helped in reaching the committee’s goals. We realized that developing a charter provided all Sustainability Committee members with a common understanding of the intended outcomes. Hiring a facilitator to organize and facilitate the sustainability committee’s meetings, one who had experience in constructing effective agendas and successfully communicating, helped in achieving the meeting goals. Employing the services of an external facilitator, as opposed to a committee member, ensured all members were fully engaged. Additionally, using the services of an external facilitator allowed MDH staff to participate fully and focus on sharing their expertise and technical assistance when needed.

#### Empowering SPC Staff to Advocate for Sustainability

The numerous sustainability training courses organized by MDH empowered SPC staff to be their own advocates. SPC staff willingly spoke up and shared their stories using MSPSI progress data to make their case even though this kind of professional experience was new to most of them. For example, with some coaching from MDH, SPC staff testified successfully at legislative hearings. SPC staff not only spoke one on one with decision makers about program sustainability, but they also gave official presentations at conferences and seminars about the needs of student parents and the SPC services. Staff members collaborated across IHEs to co-write and co-present information at statewide and regional conferences. Ultimately, the SPCs’ staffs’ capacity to perform program sustainability activities increased.

#### Collecting Relevant Data that Communicate the Value of the Program

Access to data allowed SPC staff to share quantitative and qualitative information with other stakeholders, potential funders, and policy makers. Individual students sharing their personal experiences also helped promote the public’s awareness of SPCs. Focusing the evaluation design to reflect the required grant measures and those important to SPCs and the Sustainability Committee allowed the data collection to support the goals and demonstrate the value of the student parent programs.

#### Making the Database Available for Data Collection

In addition to allowing staff to enter individual data in an application that stores, records, and reports on the SPCs’ work, the database reduced administrative burden by enabling easy retrieval of and updates to an individual participant’s information (e.g., graduation and exit). MDH’s decision to support the IHEs’ individual and unique program sustainability strategies by sharing the database with SPCs proved to be invaluable. Distributing the database infrastructure and the software application to IHEs guaranteed that the database was available to collect and store data by SPCs after the grant ended. Sharing the database infrastructure with the SPCs ensured their ability to collect data, store data, and communicate the progress of the programs with potential private and public funders and decision makers.

## Limitations

This article describes lessons learned from the implementation of the MSPSI sustainability process. The lessons we shared are real-life implementation experiences peculiar to the MSPSI sustainability process, so the findings may not be necessarily generalizable to implementers in other settings.

## Conclusion

The MSPSI sustainability process demonstrates the importance of leveraging multiple community partners in securing local funds once federal funding ends. We found that getting buy-in on strategies to sustain MSPSI from multiple stakeholders serving the PSSP population, such as state agencies and the colleges and universities who operated SPC early in the program implementation phase, was critical in securing state and university funds. All nine SPCs benefited from early sustainability planning as well as relationships with multiple agencies and organizations serving expectant and parenting youth. These relationships contributed to MOHE’s decision to allocate state funds to maintain operations at eight SPCs immediately after federal funding ceased in November 2017. Even though MOHE funding was available for all SPCs, one SPC did not receive this funding because it had already secured funding from the university. As of June 2019, at least four of the funded SPCs creatively secured alternative funding through the state, colleges, and universities to maintain some components of their SPCs’ activities and services. Although the lessons learned from the field are specific to MSPSI, they can be beneficial to the broader federally funded adolescent health programs seeking diverse financial opportunities.

Adolescent health programs should start sustainability planning early in the implementation planning phase and bring together multiple agencies and organizations, policy makers, and stakeholders with interests in promoting adolescent health and well-being. Student parents have several unique needs that one institution cannot address. Linking student parent programs with a broader network of state and local organizations and policy makers will raise the public’s awareness about the benefits of these programs. Given the limited research on how to sustain programs, the lessons learned provide useful guidance on how to leverage community partners to sustain adolescent health programs, particularly student parent programs.
